# Assessment of Properties and Microstructure of Concrete with Cotton Textile Waste and Crushed Bricks

**DOI:** 10.3390/ma16206807

**Published:** 2023-10-22

**Authors:** Nastasia Saca, Lidia Radu, Roxana Truşcă, Răzvan Calotă, Daniela Dobre, Ilinca Năstase

**Affiliations:** 1 Faculty of Roads, Railways and Bridges, Technical University of Civil Engineering, 020396 Bucharest, Romania; 2 Faculty of Engineering in Foreign Languages, University Politehnica of Bucharest, 060042 Bucharest, Romania; truscaroxana@yahoo.com; 3 Building Services Faculty, Technical University of Civil Engineering, 020396 Bucharest, Romaniailinca.nastase@utcb.ro (I.N.); 4 Faculty of Civil, Industrial and Agricultural Buildings, Technical University of Civil Engineering, 020396 Bucharest, Romania; daniela.dobre@utcb.ro

**Keywords:** concrete, cotton textile waste, crushed brick, waste valorization, properties, microstructure

## Abstract

Cotton textile waste (CW) and crushed bricks (CB) are wastes generated by the textile and construction industries that cause adverse effects on the environment. This paper explores the effect of adding 1, 2, 5, and 10 wt.% of CW and CB, instead of natural sand under 1 mm (50 to 100 vol.%), on the properties of concrete. The study included the analysis of workability, density, water absorption, thermal conductivity, mechanical strengths, and electron microscopy. The results show that the presence of CW and CB increased the water required to obtain the same slump value as reference, R. Concretes with CW provided better performance in terms of density, water absorption (for 1 wt.%), and splitting strength (for 1 to 2 wt.%). The 28 days of compressive strength decreased with increasing CW (33.3 MPa for R and 26.9 MPa for 2 wt.% of CW). The partial substitution of sand decreased the workability and density and increased the mechanical strength of concrete. The presence of both CW and CB decreased workability, density, and mechanical strengths. Regarding the ability of concrete to transfer heat, the addition of CW and CB decreased the thermal conductivity value (e.g., 0.32 W/(m·K) for 1 wt.% of CW compared to 0.37 W/(m·K) for reference).

## 1. Introduction

Globally, the depletion of natural resources continues to accelerate while huge quantities of waste are still piling up [1]. Each year, the construction sector uses more than 3 billion tons of raw materials [2]. At the international level, great efforts are being made to build a more sustainable economy through the transition from a linear economy to a circular economy. At the national scale, Romania’s sustainable development strategy for 2030 includes the transition to a circular economy [3]. By increasing construction productivity and adopting circular economy concepts, we can reduce waste and save more than USD 100 billion annually [2]. At present, the influences of each type of waste (construction and demolition wastes and textile wastes) on the properties of concrete are studied. Some research outcomes in the study of concrete with textile wastes and concrete with crushed bricks wastes are presented below.

### 1.1. Textile Wastes in Concrete

Due to the rapidly evolving fashion industry, there is a steady increase in the production of textile fibers, textile consumption, and textile waste [4]. Over 9.5 million tons of textiles are used in Europe each year, of which 5.6 million tons are discarded. The combined municipal solid waste stream contains more than 4 million tons of textile waste that are not collected separately [5]. Most post-industrial textile waste is burned or dumped. The open dumping of textile wastes pollutes the environment, contaminates the groundwater, and generates greenhouse gases. Degradable textiles break down into methane, while organic fabrics, like wool, break down into ammonia [6]. Synthetic non-biodegradable fabrics build up in landfills, posing environmental and societal problems [7]. Turning waste into resources is the biggest challenge and a major concern in present society. Sorting the waste textiles is a difficult problem and can be solved with non-destructive sorting technology using artificial intelligence technology [8]. By combining a self-developed online near-infrared device with artificial intelligence technology, Du et al. created an online near-infrared (NIR) spectrum library that provides identification models for more than 13 different categories of waste textiles [8]. Valorizations of textile wastes, especially textile waste fibers, in the building construction field have become a great area of interest in recent research activities. By using unidirectional glass fiber preform and needle punched jute nonwoven fabric for lamination, Kamble and Behera [9] developed new hybrid composites reinforced with a carded web of cotton fibers recovered from discarded cotton textiles. Compared to composites reinforced with cotton web, the results showed that those containing 14% weight of jute nonwoven had enhanced tensile and flexural strengths and Izod impact by 28, 21, and 99%. The results of Sadrolodabaee et al. [10] on the influence of short textile waste fibers on the mortar properties showed that those fibers may be used as potential reinforcement in building materials, with 8% being the ideal amount.

A novel technique for creating sound insulation materials was investigated by Dissanayake et al. [7] using waste cotton/polyester mixtures and natural rubber as bonding agents. The authors found the parameter process of producing insulation panels (15 tons and 150 °C). The panels had a noise reduction coefficient between 0.5 and 0.7. Selvaraj and Priyanka [11] studied the influence of synthetic and semi-synthetic cloth pieces cut into small pieces on concrete’s mechanical strengths and impact resistance. The results showed an increased energy absorption and flexural strength of concrete with cloth content from 1 to 5%. Increases in acoustic insulation and decreases in compressive strength were observed by Anglade et al. [12] for concrete with polyester fabric (30 mm × 60 mm, thickness of 2 mm). Patnaik et al. [13] discovered that the thermal conductivity of waste with 100% recycled polyester fiber is 9.4% higher than waste with 100% woolen, and the thermal conductivity is also decreased. Briga-Sa et al. [14] also achieved improved thermal insulation qualities when creating cementitious materials with textile waste with a maximum length of 3 cm (70% wool, 25% viscose, and 5% elastane). The results showed that blocks with 6.25%, 8.16%, and 8.75% textile waste are suitable for thermal insulation properties (thermal resistance of blocks was 0.34 m^2^ K/W, 0.61 m^2^ K/W, and 0.67 m^2^ K/W).

### 1.2. Crushed Brick Waste in Concrete

Construction made up 37.5% of the total waste generation in the EU in 2020 [15]. As a consequence, construction and demolition are priority areas in the EU according to the Circular Economy Action Plan (EC 2015) and are the subjects of numerous research activities. Construction and demolition wastes consist of plastic, concrete, excavated soil, bricks, glass, wood, polymers, metals, ceramic materials, etc. [16]. In recent years, waste clay bricks were sorted, screened, and crushed in order to obtain different particle sizes for the construction industry, as shown in numerous scientific papers. Crushed brick waste is used as alternative materials, like recycled fine aggregates [17], recycled coarse aggregates [18], and supplementary cementitious material [19]. Brick wastes are also studied as a powder in geopolymers. The use of clay brick waste powder as ceramic waste aggregates in mortar has been looked into by Lam et al. [20]. The results showed that brick powder has pozzolanic activity; increasing the brick powder content of mixtures decreases drying shrinkage and flow spread. The substitution of cement with brick powder resulted in decreased early compressive strength. Adding powders from construction and demolition wastes decreases the workability of the concrete mixture because of the high water demand for waste [21,22]. Sai Rahul et al. [23] observed that crushed bricks may be used as substituents of fine aggregates (between 150 µm and 4.75 mm) up to 75% and aggregates <150 µm limited to 10%, in paver blocks. Dang and Zhao [24] studied the influence of waste clay bricks content as fine aggregates (0%, 25%, 50%, 75%, and 100%) and additional water volumes on the properties of concrete. Concrete with no additional water or with little additional water within a 50% replacement ratio had compressive strengths comparable to standard concrete. The microscopic findings showed that recycled brick concrete had increased pore content with diameters greater than 100 nm and that the interfacial transition zone had been compacted. Vieira et al. [25] observed that mixes with crushed bricks required an increasing quantity of water in order to maintain the same workability, and there was a compressive strength loss of 9.6% when fine natural sand was substituted with finely crushed bricks. Also, it was found that increasing the fine crushed bricks’ aggregate content led to increased water absorption and decreased chloride ion migration compared to the reference. Siddique et al. [26] reported that fine ceramic aggregates increased the mechanical strength and enhanced the production of hydration products.

Cachim [27] stated that for a 15% replacement of natural coarse aggregates, brick waste could be utilized as a partial replacement without reducing the characteristics of concrete, and for a 30% replacement, reductions of up to 20% could be made. Rashid et al. [18] substituted natural coarse aggregates with 30% ceramic aggregates in concrete and observed an increased compressive strength. 

Based on these results, the present work aims to assess the effect of CW and CB on the performance of concrete. Cotton textile waste was chosen for this study because cotton and cellulose derivates represent 30–50 wt.% of the textile waste [28]. In this context, the article presents the influence of CW (0, 1, 2, 5, and 10 wt.%), which otherwise ends up in a landfill, and CB as partial replacements of natural sand, under 1 mm (up to 100 vol.%), on the properties of concrete. For this purpose, different concrete mixes have been investigated for their workability, water absorption, compressive strengths, and flexural and splitting strength. The microstructure of concretes has also been assessed using microscopic imaging.

**Figure 1:**
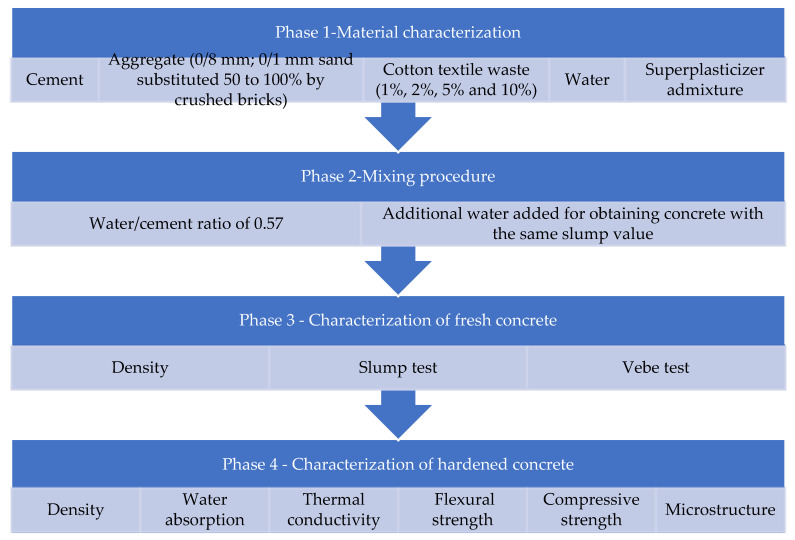
Brief presentation of the testing methodology.

The experimental program is presented below (Figure 1).

## 2. Materials and Methods

### 2.1. Materials

The cement used was a commercial cement, CEM II/A-LL 42.5 R, produced by Heidelberg Materials Romania, complying with SR EN 197-1 [29]. A natural siliceous aggregate up to 8 mm was used.

In order to obtain CB, the bricks with/without mortar were aggregated and then manually broken into more pieces to allow a better grinding process. The pieces were crushed with a jaw crusher, Retsch BB 200. The outcome dimension of the particles was modified by the change in distance between the two metallic plates. Due to the fragile nature of the bricks, the process was fast and had good efficiency. After crushing, the material was sieved to separate the particles under 1 mm. This portion of the material was used to substitute natural siliceous sand in CB concrete (50 to 100 vol.% substitution percent).

**Figure 2:**
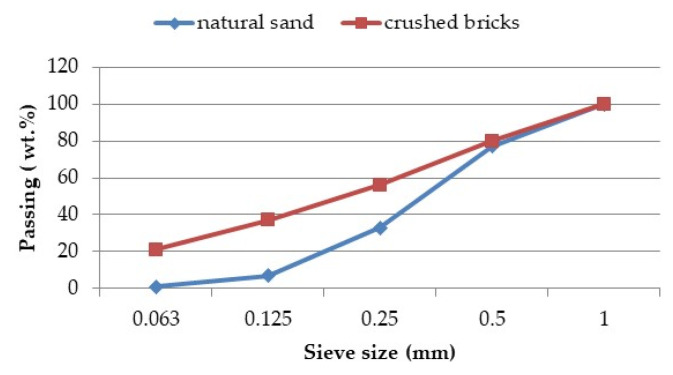
Grading size distribution of aggregates under 1 mm.

The grading curve of the natural sand and crushed bricks with a maximum size of 1 mm is presented in Figure 2.

Water absorption of CB, tested according to SR EN 1097-6 [30] was 33.7%, higher than that of the natural sand fraction 0/1 mm (18.1%). According to Cachim [27], at least 75% of the overall absorption happened during the first two minutes, and after five minutes, the proportion increased to at least 91% of the total absorption. CB water absorption is very important during concrete mixing. The density of CB fraction 0/1 mm was 2620 kg/m^3^ and of 0/1 mm sand was 2640 kg/m^3^.

**Figure 3:**
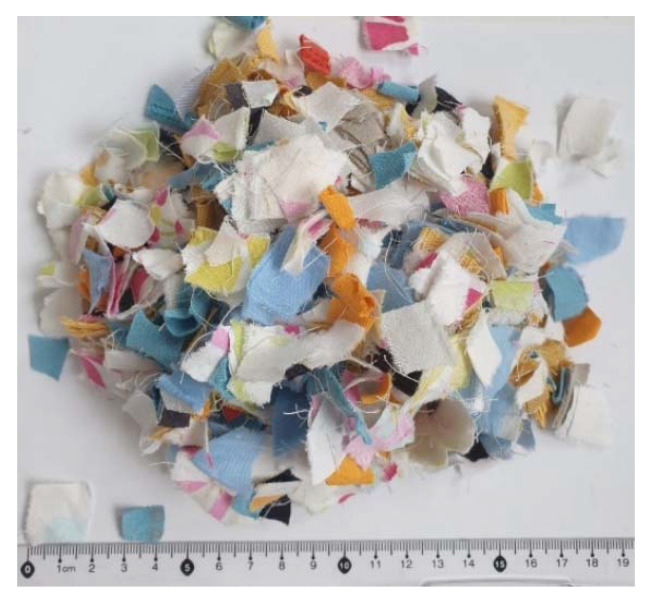
Cotton textile waste.

CW was obtained by cutting used clothes into small pieces (approximately 20 mm × 20 mm), as shown in Figure 3. The loose bulk density was 70 kg/m^3^. The tensile characteristics of fabric and yarns depend on both fiber arrangements (including length, diameter, friction, etc.) inside the yarn and fabric structure and also on the tensile properties of fibers. The tensile properties of cotton fibers are influenced by the internal structure of the fibers. Cotton fibers are not homogeneous in their physical properties and dimensions. Their maturity, diameter, and fineness is different from fiber. Sometimes, even alongside the length of a fiber, there is a variation in physical properties, such as cellulose density. According to Asim et al. [31], the tensile strength of cotton fibers is between 287 and 597 MPa. In the case of cotton fiber yarn, Chokshi et al. [32] obtained tensile strength values between 16.9 and 29.8 MPa, depending on the cross-head movement rate (up to 20 mm/min).

By testing some samples from cotton fabrics used in the research, the tensile strength values between 13 and 43 MPa were obtained.

A polycarboxylate superplasticizer, Viscocrete 2320, produced by Sika Romania, was used in the proportion of 1.8% with weight of cement. The admixture has a density of 1.042 ± 0.02 g/cm^3^, pH = 4.5, chloride content < 0.1%, and alkali content in the form of Na_2_O < 0.5%.

### 2.2. Concrete Mix Design

The evaluation of the concrete qualities with bricks and textile waste content was the primary goal of the experimental activity. The reference concrete’s mix ratio was developed using SR EN 206 + A2 [33] and NE 012 [34], as shown in Table 1. The reference concrete had a water/cement ratio (w/c) of 0.57. To create concrete with the same slump value, more water is added to the concrete mix, w_a_, and the water/cement ratio increases with the value of w_a_/c. Natural sand under 1 mm was substituted with CB at 50% and 100% replacement levels by volume. The textile wastes were added in proportion to 1, 2, 5, and 10 wt.% of the weight of cement.

### 2.3. Mixing Procedure

The following subsection describes how the concrete was produced. The dry weight of the concrete components was first measured in accordance with the mix design. Then, for 60 s, the aggregate and textile wastes were mixed. The mixer was filled with 30% water, and it was blended for 60 s. The mixer was filled with cement, which was then stirred for 60 s. The remaining water and superplasticizer were added to the mixer gradually while being stirred for 180 s to ensure uniformity.

The vibrating table compacted the concrete after it had been cast into the mold, and it was then left at room temperature for 24 h. The concrete was removed from the mold and put in a typical curing environment with saturated humidity until it reached the testing age.

### 2.4. Testing Methods

#### 2.4.1. Concrete in Fresh State

The workability of fresh concrete was tested using slump and Vebe tests in compliance with SR EN 12350-1 and SR EN 12350-3 [35,36]. The fresh concrete density was tested according to SR EN 12350-6 [37], using a 5 L container, a vibrating table, and a balance with an accuracy of 0.01 kg.

#### 2.4.2. Density of Hardened Concrete

The density of 28-day-old concrete was tested according to SR EN 12390-7 [38]. The volume of three samples (cube 100 mm × 100 mm × 100 mm) for each concrete mix was calculated using measurements made with a caliper capable of determining the dimension of the specimen within 0.5%. The samples were weighted as received, with an accuracy of 0.01% of the mass of the specimen.

#### 2.4.3. Water Absorption Due to Capillary Action

The test was performed on three prismatic specimens measuring 40 mm × 40 mm × 160 mm for each mix. After curing for 28 days, the specimens were dried in an oven at 105 ± 5 °C until a constant weight was recorded, *m_d_*. The specimens were then cooled in an exicator. The dimensions of the faces immersed in water were measured, and the gross area, *A_s_*, was calculated. The lateral surfaces were sealed in order to ensure one-directional water flow through the samples. The samples were weighted and immersed in water (the immersed depth was kept at 3–5 mm). *At* different time intervals, *t*, (4, 9, 25, 64, and 100 min), the concrete specimens were taken out of the tray, the surface water was wiped off, and the mass change was recorded, *m_s_*. Then, the samples were re-immersed. The test was performed based on SR EN 772-11 [39]. The water absorption has been calculated with Equation (1).

(1) $$c_{w}=\frac{m_{s}-m_{d}}{A_{s}.t}\cdot 10^{6}\;(g/m^{2}\cdot s)\;$$

#### 2.4.4. Mechanical Strengths

The compressive strength was tested according to SR EN 12390 [40] on 100 mm cubes using a WPM 3000 kN hydraulic testing machine. Before loading, the samples were positioned perpendicular to the casting direction. The concrete was evaluated for compressive strength at the ages of 2 and 28 days on three samples for each term.

Splitting strength was conducted at 28th day on three concrete cubes with dimensions of 100 mm × 100 mm × 100 mm, using a WPM testing machine with a maximum load capacity of 300 kN. The flexural strength (one-point loading) was tested on three prismatic samples (40 mm × 40 mm × 160 mm) after 28 days, using the same machine that was used in the splitting test.

#### 2.4.5. Thermal Conductivity

The thermal conductivity of concrete was tested according to EN 12664 [41]. The material’s thermal conductivity was determined using a Heat Transfer Service Unit for Building and Insulating Materials, type H111N. The specimen was placed between a hot plate and a heat flowmeter attached to a cold plate. A handwheel loading mechanism allows the test specimen to be pressed between the two plates. The material thickness can be measured with high accuracy. The material is pressed between hot and cold plates using a handwheel loading mechanism connected to the control panel. When the optimum position is set, a lamp turns off, and the thickness evaluation can be conducted. Due to the high precision of the sensors, the precision is between ±2%, resulting in a high-accuracy measurement. The entire unit is well insulated, and the measurements were recorded when the average temperature corresponding to each plate remained constant, or, in other words, when a steady-state regime was attained.

The hot plate temperature is controlled using a PID controller to a set value. Since the cold plate temperature is directly dependent on the cold-water temperature from the distribution network and cannot be modified, the hot water temperature is manually set so that a temperature difference of 15–20 K is recorded between the two plates.

The thermal conductivity depends on both plates’ average temperatures, heat flowmeter output, and six calibration constants that depend on the apparatus.

#### 2.4.6. Structure of Concrete

To acquire a better understanding of the macroscopic properties of concrete, microscopy tests were also carried out. The morphology of reaction products was studied via SEM analysis recorded on a SEM/EDAX High Resolution Scanning Electron Microscope, Quanta Inspect F FEG (resolution 1.2 nm) with EDAX (133 eV resolution at MnKα)—FEI Company. Prior to imaging, a small layer of conductive gold was applied to the samples. The chosen concrete was 28 days old.

## 3. Results and Discussion

### 3.1. Workability of Concrete

**Figure 4:**
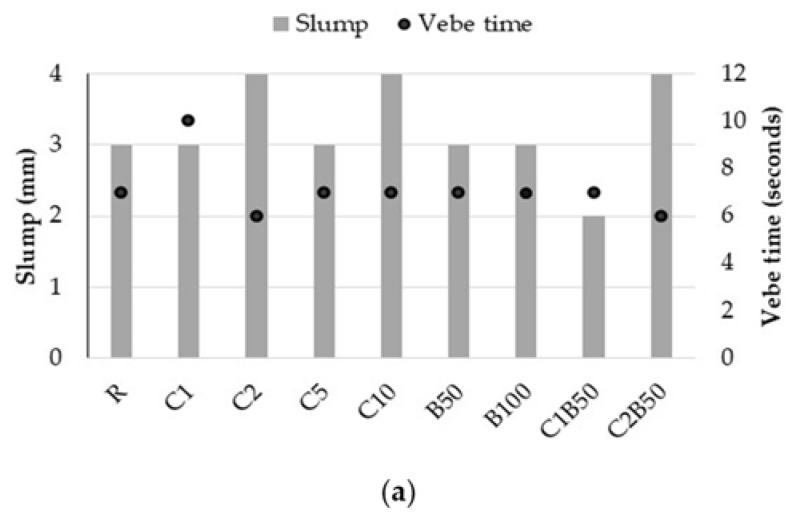

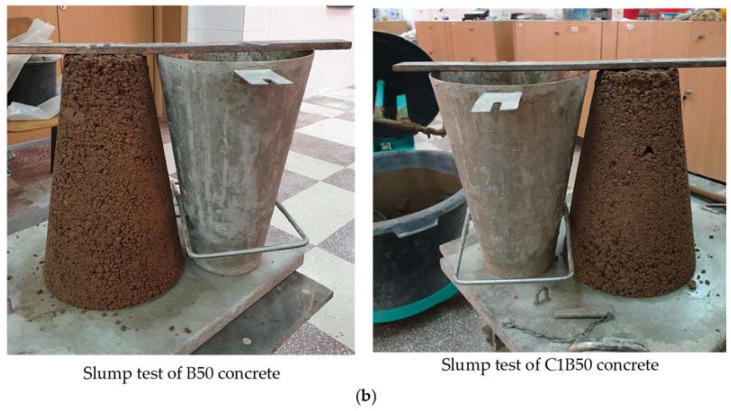
Slump and Vebe time of concrete (**a**) and images of the concrete during the slump test (**b**).

The workability of concrete was measured with the slump test and the Vebe test. The average slump of concrete was 3 mm, as shown in Figure 4. According to ACI 116R-90, no-slump concrete has a slump of less than 6.35 mm [42]. For the purpose of maintaining the workability approximately constant, additional water was added, as shown in Table 1.

The Vebe test measures the consistency of concrete through the time in which the concrete changes from the truncated shape to the cylindrical shape under the effect of vibration. The Vebe time was between 5 and 10 s. It is clear that utilizing CB as a substitute for natural sand reduces the workability of concrete and necessitates the use of more water, depending on the content of CB. The concrete B100 (0/1 mm sand substituted with CB) needs more water than the concrete B50 in order to obtain the same slump. This is a consequence of the angular and irregular shapes of CB compared to the rounded and smoother particles of natural aggregate, which raises the friction between particles and affects the concrete’s workability [43]. As a consequence, a substitution of 50% of natural sand under 1 mm with CB was selected for concrete with textile waste.

The water quantity added to concrete with 1% and 2% textile waste is slightly higher than that of the reference and smaller than in the case of concrete with crushed brick content. The addition of textile waste by up to 10% led to a significant increase in the necessary water to achieve the same slump of concrete.

The presence of both textile waste and CB led to a significant increase in water added for obtaining concrete in comparison to reference, as a consequence of the water absorption of waste.

### 3.2. Density of Fresh and Hardened Concrete

**Figure 5:**
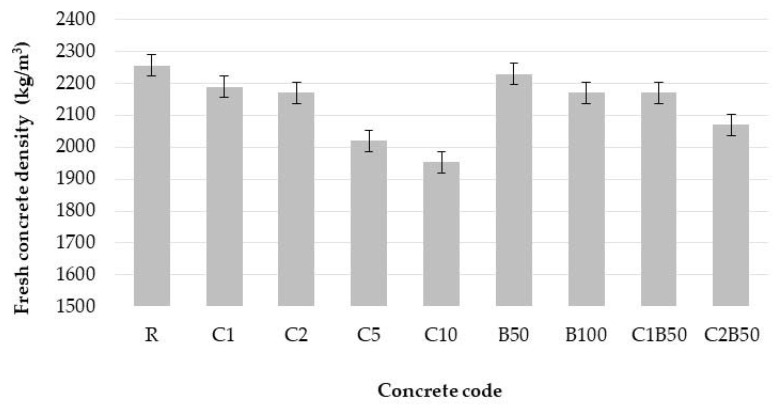
Density of fresh concrete.

The results exhibit an obvious reduction in the density of fresh concrete when crushed bricks and textile wastes are used (Figure 5). The replacement of natural sand under 1 mm with crushed bricks decreased the density by 1.2–3.8% in comparison to reference R.

The density of fresh concrete with textile waste was lower than the reference. Increasing the textile waste content decreased the density of fresh concrete; the minimum value was for concrete with 10% cotton waste (~1950 kg/m^3^). The presence of both kinds of wastes led to a decrease in density by 3.8–10.5% in comparison to reference; the density values were from 2070 to 2170 kg/m^3^.

**Figure 6:**
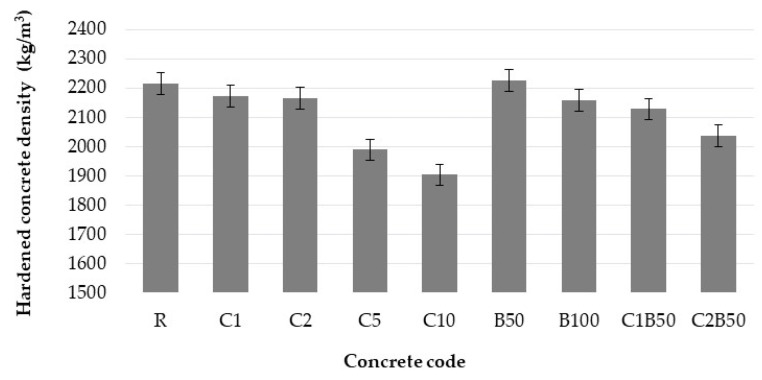
Density of hardened concrete (28 days).

The density of 28-day-hardened concrete varied from 1965 kg/m^3^ to 2216 kg/m^3^ (Figure 6). The presence of waste decreased the density of concrete, which hardened for 28 days.

A decrease in density was expected due to the higher water quantity required to obtain concrete with the same slump value as R and lower density of CW and CB.

### 3.3. Water Absorption Due to Capillary Action

**Figure 7:**
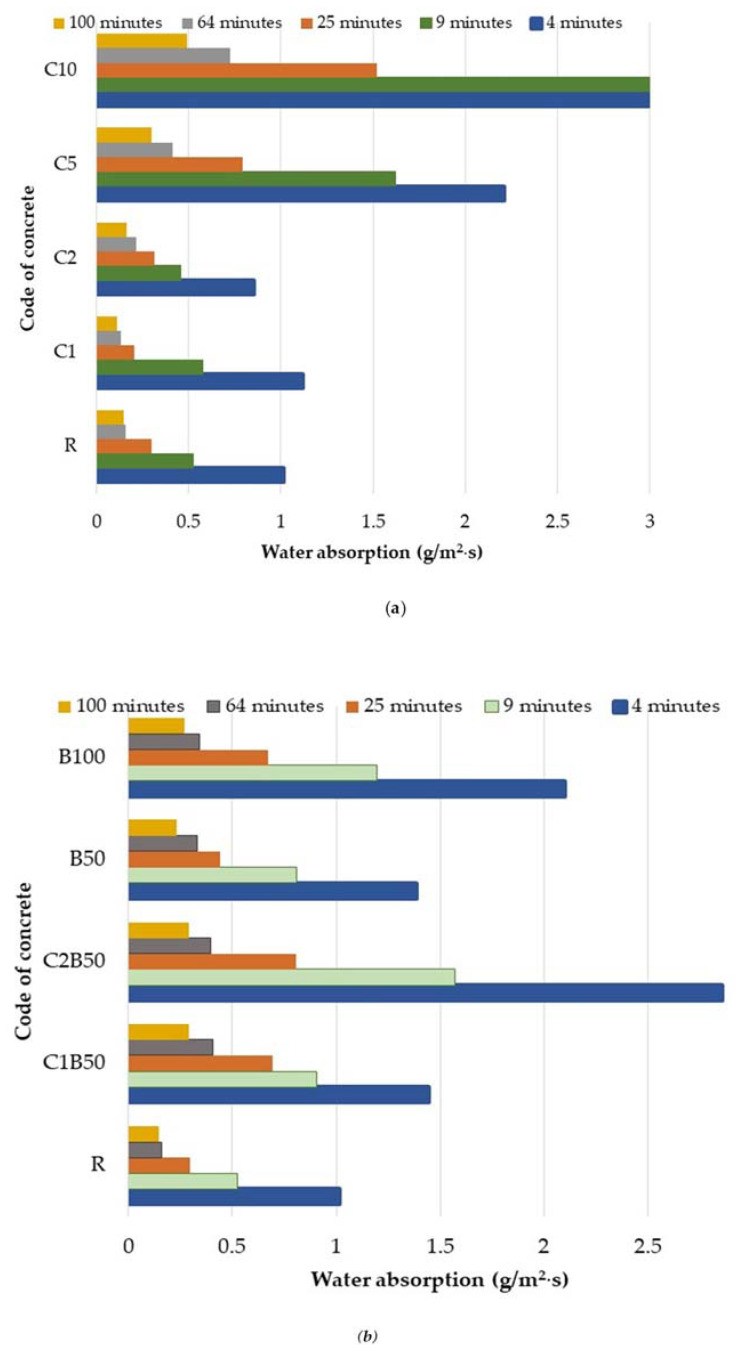
Water absorption of concrete with cotton waste (**a**) and brick (**b**).

The results of capillarity can be seen in Figure 7. Specimens containing CW exhibited higher water absorption values compared to R. This was to be expected given the high w/c ratio (including additional water) required to retain the same workability as the R. After 100 min, C1 had water absorption decreased by 24% as compared with R. Increasing the cotton waste content led to higher water absorption (0.49 g/m^2^·s for C10, compared to 0.30 g/m^2^·s for C5, 0.16 g/m^2^·s for C2, and 0.11 g/m^2^·s for C1 after 100 min).

For both B50 and B100 mixes, a higher quantity of additional water led to increased water absorption. Compared to R, the replacement of natural sand with CB increased water absorption by 61% and 81% for 100 min, as shown in Figure 7b. The unused water in the cement hydration, which was simple to make into a workable mix, led to a larger capillary pore system.

Concretes with both CW and CB consistently absorbed more water than C1 and C2. Higher cement matrix porosity is most likely a result of the higher water content of concrete. The water absorption decreased in the C1B50 > B50 > R and C2B50 > B50 > R series. Higher water absorption for concrete with recycled brick aggregate was also obtained by [24] and attributed to the internal microcracks and porous structure of the aggregate.

### 3.4. Thermal Conductivity

The thermal conductivity test is significant for assessing the ability of concrete to transfer heat. Thermal conductivity, λ, was measured for selected concretes on slabs. The concrete with 1% CW had a λ of 0.32 W/(m·K). The presence of both CW and CB improved the thermal insulation ability of concrete (e.g., 0.35 W/(m·K) for C1B50 compared to 0.37 W/(m·K) for R). The concrete B50 had a λ of 0.35 W/(m·K). The lower λ values of concrete with textile wastes, in comparison to R, were consequences of the fact that textile waste has a conductivity value that is more than 10 times lower than that of concrete. Cotton’s limited ability to effectively transport heat is due to its poorer thermal conductivity (0.026–0.065 W/(m·K)) than many synthetic and other natural fibers [44,45]. Furthermore, the concrete with cotton has a higher porosity than the reference. The water absorption of these concretes had high values, which indicates increased open porosity.

### 3.5. Mechanical Strengths

The mechanical strength of concrete is presented in Table 2. Results showed that concrete with CW had a smaller compressive strength than reference concrete after 28 days. The decreased compressive strengths can be a result of a reduction in cohesiveness in concrete as a consequence of the presence of textile waste. Also, the hydrates seem to be partially formed on the textile wastes. Mix C2 gains over time compressive strength to a point comparable to C1 and R. Table 2 shows a gradual reduction in 28-day compressive strength when textile waste content increased from 2 to 10%.

For 50% substitution of natural sand under 1 mm with CB, it was observed a reduction in strength for 2 days and an increase for 28 days, compared to the reference (33.7 MPa for B50 and 33.3 MPa for R). CB seems to contribute to the densification of the concrete structure due to a higher content of fine particles (0.125 µm sieve passes of 37% for CB and 7% for natural sand), and the rough texture and angular shape of CB led to a denser interfacial transition zone between the ceramic aggregate and matrix. When the substitution percent of natural sand under 1 mm increased to 100%, compressive strength decreased at 28 days; the reduction was 22% compared to the reference and 23% in comparison to B50. The mixes B50 and B100 stand out for a significant increase in compressive strength over time compared to other mixes. Similar results were obtained by [25] and explained as a result of a possible pozzolanic reaction between silica and alumina from crushed brick aggregates and Ca(OH)_2_.

The concrete with both CW and CB had a lower compressive strength than the concrete with only textile waste and reference, the decrease being significant for 2% textile waste. The decrease in compressive strength was 18…33.5% in comparison to concrete with textile waste (no bricks) and 35.1…49.3% compared to B50.

In the case of flexural strength, the reference concrete had a value of 7.09 MPa (28 days), which represents 21.3% of compressive strength. The addition of textile wastes increased the ratio between flexural strength and compressive strength and slightly decreased the flexural strength compared to the reference. The concrete with the highest flexural strength was B50 (22.3% bigger than R). A complete substitution of natural sand under 1 mm with CB reduced the flexural strength by 30% compared to B50.

The splitting strength of concrete with 1% and 2% textile waste was higher than R. The explanation for the increase in splitting strength is that cotton textile waste acts as reinforcement.

The substitution of natural sand with CB had a positive effect on the flexural and splitting strength, probably due to the particle size distribution of CB and the pozzolanic activity of CB.

**Figure 8:**
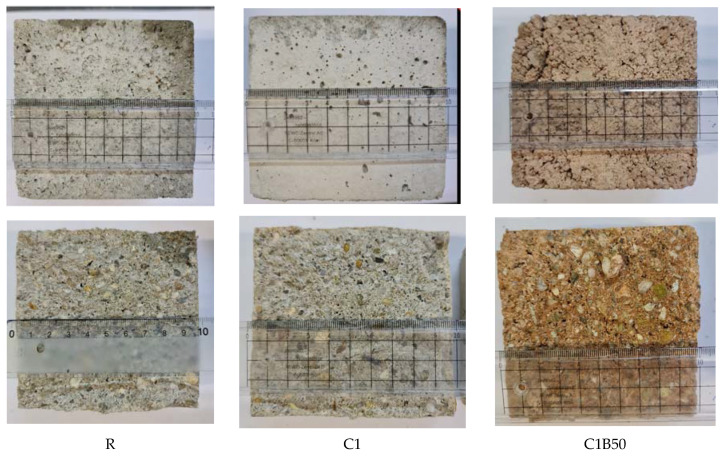
Image of some specimens before and after testing (cubes 100 mm × 100 mm × 100 mm).

Figure 8 shows the split view of some cubes after the splitting strength test.

Based on the values from Table 2, the ANOVA analysis shows that there is a statistically significant difference between the means of compressive strength at 2 and 28 days (F(1, 26) = [13.576], *p* = 0.001, and F_crit_ = 4.225). On the other hand, the relationship between the compressive strength (MPa) and the CW content (%) is presented in Table 3, in the form of matrix correlation with Pearson coefficients. The negative correlation coefficients show an inverse linear relationship between the considered variables, implying that an increase in one variable (5% and 10%) is characterized by a decrease in the other.

Based on the data obtained experimentally, it is found that the thermal conductivity correlates with only the splitting strength (R^2^= 0.79, i.e., 79% of the thermal conductivity variation is explained by the variation of the splitting strength; the *p*-value is below 0.05). Also, the regression line from a linear regression analysis turns out to be the following:

Thermal conductivity = 0.56 − 0.089 × Splitting strength

(If splitting strength increases, thermal conductivity decreases.)

### 3.6. Microstructure

**Figure 9:**
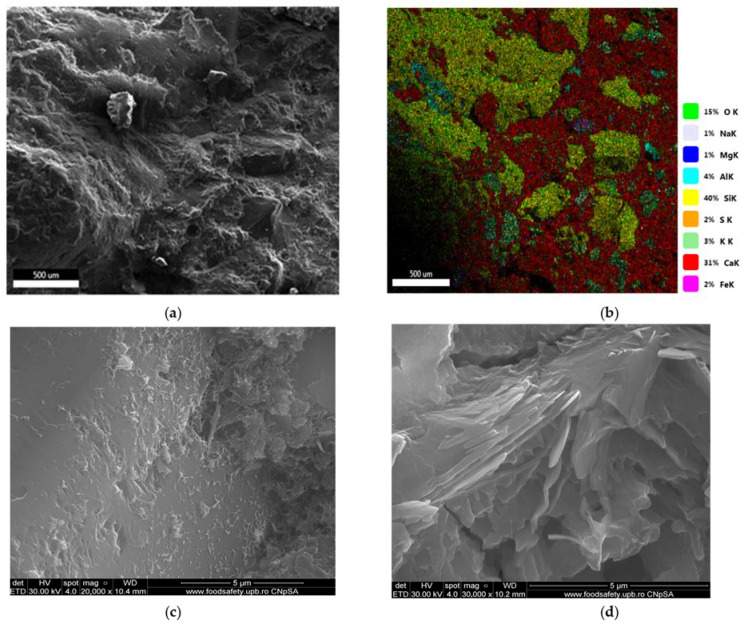
SEM images (**a**,**c**,**d**) and EDAX map (**b**) for R.

**Figure 10:**
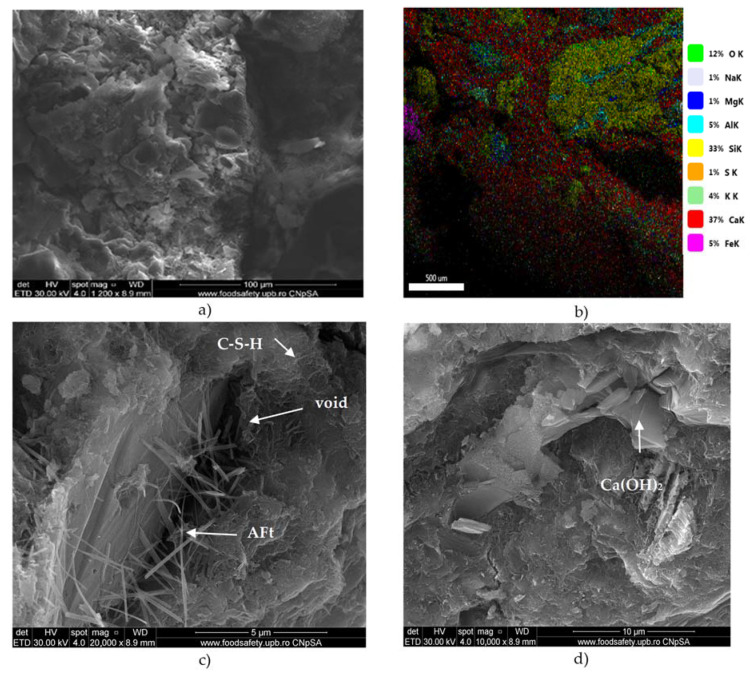
SEM images (**a**,**c**,**d**) and EDAX map (**b**) for B50.

**Figure 11:**
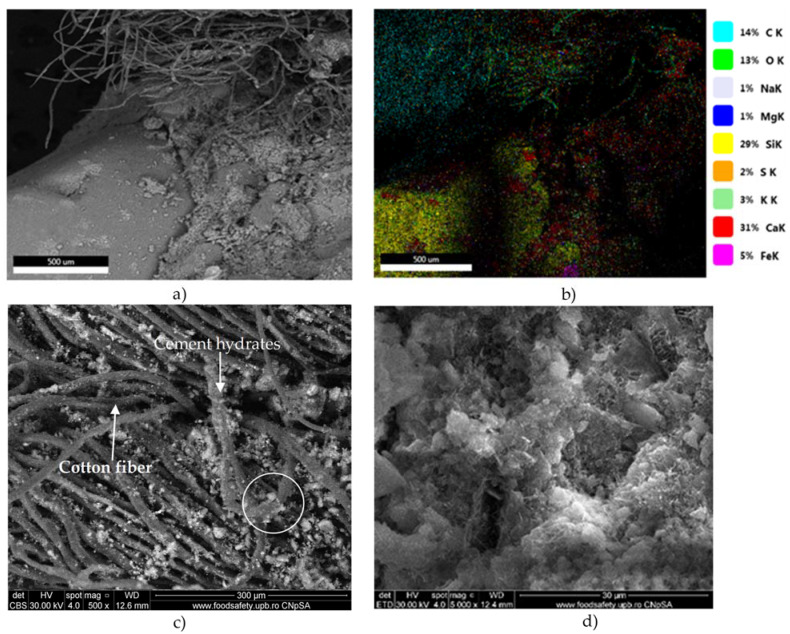
SEM images (**a**,**c**,**d**) and EDAX analysis (**b**) of C1B50 concrete.

The SEM images of concrete R, B50, and C1B50 are presented in Figure 9, Figure 10 and Figure 11. The age of the concrete was 28 days. The presence of hydrates can be seen, specific to the cement matrix–low crystallized calcium silicate hydrates, needle-like crystals of ettringite (bigger around the voids), and Ca(OH)_2_ hexagonal crystals. Figure 9 showed plate-like formations, rich in Ca and O, probably calcium hydroxide impure with Si, Al, Mg, and Fe (EDAX analysis showed 59.88% O, 23.57% Ca, 10.64% Si, 2.91% Al, 1.62% Mg, and 1.32% Fe).

Partial substitution of natural sand with CB led to an improvement in the microcrystalline aspect of concrete B50 compared to R. Images from Figure 10 highlighted the presence of C-S-H as crumpled sheets and AFt phases. According to [24], concrete with crushed brick aggregates has a denser interfacial transition zone than concrete with natural aggregate. This is due to the more tortuous interface between ceramic aggregate and cement matrix, supplementary hydrates brought in by the pozzolanic reaction, and the porous character of brick aggregate, which absorbs internal free water in the mix.

Figure 11 proves the microcrystalline aspect of concrete C1B50 and the presence of voids. The fibers from cotton textile waste are wrapped or embedded in the cement matrix. The EDAX analysis of the area marked with a circle in Figure 11c showed that the phases on the cotton textile waste contained mainly O and C but also Ca, Si, Fe, K, Al, and S, which suggests the presence of precipitation products (atomic %: 3.2% Si, 0.7% Al, 12.14% Ca, 3.44% Fe, 24.36% C, and 54.58% O).

## 4. Conclusions

This paper presents an experimental study on the effect of cotton textile waste (CW) and crushed bricks (CB) on the properties of concrete. The experimental data led to the following conclusions:

1. The addition of cotton textile waste in concrete (1, 2, 5, and 10 wt.%) has led to increased water quantities required for obtaining concrete with the same slump value as reference, due to the absorption capacity of waste. The same trend was observed for concrete with CB (50 to 100% CB substituted the natural sand under 1 mm) and concrete with both wastes. The additional water-influenced properties were tested. The density of fresh and hardened concrete decreased compared to the reference;
2. The water absorption via capillarity increased for mixes with wastes because of the increased water/cement ratio, which led to a more porous cement matrix;
3. The 28-day compressive strength of concrete with 1 and 2 wt.% CW was between 26.7 and 26.9 MPa, representing a decrease of 19.2% and 19.8%, respectively, compared to the reference. The compressive strength of concrete with 10 wt.% significantly decreased to 8.8 MPa, compared to 33.3 MPa for reference. The same observation is also valid for flexural strength. Concretes with 1 and 2 wt.% CW had higher splitting strength than reference due to their reinforcement effect;
4. The partial substitution of natural sand under 1 mm with crushed bricks (50 vol.%) led to the 28 days’ mechanical strengths comparable to reference (compressive strength of 33.7 MPa compared to 33.3 MPa). The increase in CB concrete compressive strength over time was significant over time compared to other mixes, which suggests a pozzolanic reaction. When CB content increased to 100 vol.%, the mechanical strengths decreased;
5. The incorporation of both CW and CB in concrete resulted in a decreased compressive and flexural strength, except for the splitting strength;
6. From a statistical point of view, the conclusion stated above regarding the diminishing of mechanical strength with the increase in the addition of textile waste is supported by correlation coefficients lower than zero;
7. The SEM and EDX analyses showed an improved microcrystalline aspect of concrete with crushed brick and the presence of hydrates on the CW;
8. The addition of cotton textile waste in concrete leads to a decrease in the thermal conductivity to 0.32 W/(m·K) compared to 0.37 W/(m·K) for reference, improving the insulation properties, which are directly related to the buildings’ energy efficiency.

The use of CW in concrete seems to be a viable solution with a positive impact on the environment, reduced greenhouse gas emissions, and minimized the landfill disposal through the successful use of these waste materials in construction. Further studies are necessary, especially aimed at the influence of the size of cotton waste on the properties of fresh and hardened concrete.

## Figures and Tables

**Table 1:** Mix proportion of concretes.

Concrete Code	w/c Ratio	Cement (kg)	Coarse Aggregate (kg)	Sand Aggregate (kg)	Crushed Bricks (kg)	Textile Waste (kg)	w_a_ */Cement
R	0.57	262	629	1336	0	0	0
C1	0.57	262	629	1336	0	2.62	0.011
C2	0.57	262	629	1336	0	5.24	0.011
C5	0.57	262	629	1336	0	13.1	0.130
C10	0.57	262	629	1336	0	26.2	0.149
B50	0.57	262	629	992	344	0	0.160
B100	0.57	262	629	649	687	0	0.449
C1B50	0.57	262	629	992	344	2.62	0.171
C2B50	0.57	262	629	992	344	5.24	0.172

* w_a_ is additional water added to obtain concrete with the same slump value.

**Table 2:** Mechanical strengths of concrete.

Code of Concrete	Compressive Strength (MPa)		Flexural Strength (MPa)	Splitting Strength (MPa)
	2 Days	28 Days	28 Days	28 Days
R	19.9	33.3	7.09	2.25
C1	18	26.7	6.02	2.71
C2	20.5	26.9	6.13	2.95
C5	10.4	17.5	2.55	1.62
C10	5.9	8.9	1.92	1.23
B50	8	33.7	8.67	3.11
B100	8	26	6.05	2.99
C1B50	13.2	21.9	5.95	2.36
C2B50	10.2	17.9	5.74	2.29

**Table 3:** Matrix correlation for mechanical strength (MPa) and the CW content (%).

	Percent Cotton (%)
Compressive strength	0.980
Flexural strength	0.931

## Data Availability

Not applicable.
